# The Pathological Society of Philadelphia

**Published:** 1859-11

**Authors:** 


THE PATHOLOGICAL SOCIETY OF PHILADELPHIA.—At a meeting held on the 14th of October, the following officers were elected for the ensuing year:—

| | |
|---|---|
| *President,* | DR. STILLÉ. |
| *Vice-Presidents,* | DRS. LA ROCHE and EDWARD HARTSHORNE. |
| *Treasurer,* | DR. ADDINELL HEWSON. |
| *Secretary,* | DR. DA COSTA. |
| *Assistant Secretary,* | DR. JOHN K. KANE. |

We are happy to add that the Society is in a highly prosperous condition; its meetings are well attended, and its discussions are very animated and instructive.

